# Medicaid Claims for Contraception Among Women With Medical Conditions After Release of the US Medical Eligibility Criteria for Contraceptive Use

**DOI:** 10.5888/pcd16.180207

**Published:** 2019-01-03

**Authors:** Toyya A. Pujol, Nicoleta Serban, Julie Swann, Melissa Kottke

**Affiliations:** 1 Georgia Institute of Technology, School of Industrial and Systems Engineering, Atlanta, Georgia; 2 Emory University, Department of Gynecology and Obstetrics, Jane Fonda Center for Adolescent Reproductive Health, Atlanta, Georgia

## Abstract

**Introduction:**

The US Medical Eligibility Criteria for Contraceptive Use (MEC) identified 20 medical conditions that increase a woman’s risk for adverse outcomes in pregnancy. MEC recommends that women with these conditions use long-acting, highly effective contraceptive methods. The objective of our study was to examine provision of contraception to women enrolled in Medicaid who had 1 or more of these 20 medical conditions

**Methods:**

We used Medicaid Analytic Extract claims data to study Medicaid-enrolled women who were of reproductive age in the 2-year period before MEC’s release (2008 and 2009) (N = 442,424) and the 2-year period after its release (2011 and 2012) (N = 533,619) for 14 states. We assessed 2 outcomes: provision of family planning management (FPM) and provision of highest efficacy methods (HEMs) for the entire study population and by health condition. The ratio of the after-MEC rate to the before-MEC rate was used to determine significance in MEC’s uptake.

**Results:**

Outcomes increased significantly from the before-MEC period to the after-MEC period for both FPM (1.06; lower bound confidence interval [CI], 1.05) and HEM (1.37; lower bound CI, 1.36) for a 1-sided hypothesis test. For the 19 of 20 conditions we were able to test for FPM, contraceptive use increased significantly for 12 conditions, with ratios ranging from 1.05 to 2.14. For the 16 of 20 conditions tested for HEM, contraception use increased significantly for all conditions, with ratios ranging from 1.19 to 2.80.

**Conclusion:**

Provision of both FPM and HEM increased significantly among women with high-risk health conditions from the before-MEC period (2008 and 2009) to the after-MEC period (2011 and 2012). Health policy makers and clinicians need to continue promotion of effective family planning management for women with high-risk conditions.

## Introduction

In 2010, the Centers for Disease Control and Prevention (CDC) released the US Medical Eligibility Criteria for Contraceptive Use (MEC) to guide health care providers in making evidence-based decisions on contraception. MEC focused on 20 medical conditions that present an increased risk for adverse outcomes during pregnancy, stating that long-acting, highly effective contraception methods may be the best choice for women with these medical conditions (1). Such methods include reversible options, such as intrauterine devices (IUDs) and implants, and permanent options, such as sterilization. Sole use of behavior-based methods, such as condoms, was not recommended because of their typically high failure rates.

CDC disseminated MEC guidelines through mobile applications, publications, and presentations (2). Nevertheless, a recent survey found that providers’ knowledge of MEC was low (3). Some studies of women with the 20 MEC medical conditions found low levels of use of highly effective contraception, high levels of unintended pregnancy, and provider-imposed limitations to effective contraception options (4–7).

MEC guidelines may be particularly relevant for providers who serve low-income women, including women enrolled in Medicaid. Such women are most likely to have unintended pregnancies (8) and associated medical conditions (9). In 2016 over 20% of reproductive-aged women in the United States were insured by Medicaid (10), and in 2010 Medicaid covered health care for nearly half of all US births (11). However, information comparing provision of contraception before and after MEC’s release is unavailable. The objective of our study was to examine provision of contraception to women enrolled in Medicaid who had 1 or more of the 20 MEC-highlighted medical conditions by 1) determining the provision of family planning for these women and 2) comparing the use of highly effective contraception methods in the 2-year period before MEC’s release (2008 and 2009) with their use in the 2 years after its release (2011 and 2012) to see if an increase occurred.

## Methods

### Data sources

We used Medicaid Analytical Extract (MAX) medical claims acquired from the Centers for Medicare and Medicaid Services (CMS) for the years 2008 through 2012. The MAX data set consists of individual-level claims data for all Medicaid-enrolled beneficiaries. We examined enrollees from 14 states, which accounted for more than 50% of all Medicaid enrollees in the United States: 10 southeastern states (Alabama, Arkansas, Florida, Georgia, Louisiana, Mississippi, North Carolina, South Carolina, Tennessee, and Texas) and 4 states from other regions of the country (California, Minnesota, New York, and Pennsylvania). The Southeast was chosen as a focal point because its states are similar to each other in contraception health policy (12) and spending levels for Medicaid (13), and the health rankings of these states are among the lowest in the country (14). The other 4 states chosen were highly populous states from regions of the country that have various health policies and reimbursement levels that represent differences across the United States.

We obtained approvals to perform our research from CMS and from the institutional review board of the Georgia Institute of Technology. The study infrastructure to safeguard identifiable data followed the CMS-approved data use agreement, which allows publication of results from populations of 11 or more people (eg, patients).

### Study population

We assessed the overall population of reproductive-aged women who were enrolled in Medicaid in 2008, 2009, 2011, and 2012 in all 14 states. We investigated 2 periods: the 2 years before MEC’s release (2008 and 2009) and the 2 years after MEC’s release (2011 and 2012). Our study population was a subset of the overall population and consisted of women aged 15 to 44 who had 1 or more of the 20 conditions listed in MEC (Appendix A). We did not count women more than once if they had multiple conditions. We stratified the study population by 1) age group (15–24 y, 25–34 y, 35–44 y) (15), 2) medical condition, and 3) state of residence. We obtained the age of each woman by using the date of birth in MAX’s Personal Summary table. A woman was assigned to an age group on the basis of her age at the beginning of each period (2008 and 2011).

Medical condition was defined as 1 of the 20 MEC-identified conditions. A woman with a nonsurgical MEC condition was identified as having at least 3 Medicaid claims for that condition recorded on 3 different days in the before-MEC period (2008 and 2009) or the after-MEC period (2011 and 2012) (16). The Medicaid claim could be a claim from MAX’s Other Therapy table or MAX’s Inpatient table. Diagnosis codes of the *International Classification of Disease*, ninth edition (ICD-9) were used to identify nonsurgical conditions (Appendix A) (17). Different approaches were needed to identify women with surgical MEC conditions (bariatric surgery and solid organ transplant). To identify these women, we queried the Inpatient table of the MAX data for claims that contained the corresponding surgery condition procedure codes (Appendix A). We screened for the procedure codes in the inpatient claims that occurred in the 2 periods and assigned women to the period in which the surgery occurred. When identifying patients, we considered each condition separately, to account for comorbidities.

We identified the woman’s state of residence by the state listed on her claim. This ensured that a woman was counted in each state in which she received service.

### Outcome analysis

We considered 2 outcome measures, family planning management (FPM) and highest efficacy methods (HEMs). We documented the number of women for both outcome measures for both periods and for each medical condition.

**Family planning management**. We defined an FPM claim as one containing a diagnosis code beginning with V25, the overarching code for “encounter for contraceptive management” (17). The FPM measure includes many forms of contraception claims, ranging from discussion of contraception options with the clinician to procedures, such as inserting IUDs and sterilization. We aggregated the number of women with V25 claims for each period and each condition and compared the study population with the overall population. We considered 19 of the 20 MEC medical conditions; we excluded schistosomiasis because the number of women with these conditions was less than 11.

**Highest efficacy method, aggregate and condition-level analysis. **The HEM outcome consisted of contraception claims for IUDs, contraceptive implants, and sterilizations. MEC recommends HEMs for women with high-risk conditions. We used the diagnosis codes for IUD insertion (V25.1), IUD surveillance (V25.42), and implant surveillance (V25.43) and searched through both inpatient and other therapy claims. Because of the nature of the procedure, we searched for sterilizations (V25.2) through inpatient claims only.

We calculated HEM provision for the overall population, the study population, and each medical condition, including the number of women in the HEM outcome for each condition, the percentage rates of HEM, and the results of a 1-sided test for significance, including the lower bound of a 99% confidence interval. We considered 16 of the 20 MEC medical conditions; we excluded malignant gestational trophoblastic disease, liver cancer, schistosomiasis, and solid organ transplant because the number of women with these conditions was less than 11.

### Rate analysis

Because rates for FPM and HEM use increased nationally during the years of our study, we used rates in the overall population as a scaling factor for the study population. The scaling factor was applied to the study population use rate to accurately determine the change in rates before and after the introduction of MEC.

A 1-sided exact Poisson test was used to determine whether provision of contraception increased significantly in the study population. The alternative hypothesis was defined as the before-MEC rate being smaller than the after-MEC rate. A ratio greater than 1 indicates an increase in provision; a ratio of 1.1 indicates a 10% increase in the rate.

The test statistic comparing before-MEC and after-MEC outcome measures was scaled by the rates in each of the 2 periods by the corresponding outcome measure of the overall population (Appendix B). The test procedure was applied to all conditions together and to each MEC condition separately. For the condition-level analysis, we corrected for the testing of multiple outcomes simultaneously by using the Bonferroni correction.

## Results

### Study population

Our sample consisted of more than 12 million women in 14 states who were covered by Medicaid in both study periods (Table 1). Most reproductive-aged women enrolled in Medicaid did not have claims for these conditions; less than 5% were identified as having 1 of the 20 high-risk MEC conditions. Though low, we saw an increase from 3.5% in the before-MEC period to 3.9% in the after-MEC period. More than half of the women with high-risk conditions were in the 35-to-44 age group, 53.5% in the before-MEC period and 66.9% in the after-MEC period. The 4 most common conditions made up 83% of the study population; in order of frequency, they were hypertension, diabetes, epilepsy, and HIV.

**Table 1:** Reproductive-Aged Women in Medicaid Study Population Before and After MEC, by Age, State of Residence, and Health Condition

Variable	State Population Before MEC, 2008–2009, N = 442,424	State Population After MEC, 2011–2012, N = 533,619
**Overall population, N**	12,422,899	13,597,612
**Study population^a^**	437,018 (3.5)	527,660 (3.9)
**Age^b^, y**		
15–24	69,050 (15.8)	87,797 (16.6)
25–34	134,267 (30.7)	200,909 (38.1)
35–44	233,701 (53.5)	352,833 (66.9)
**State^b^**		
Alabama	16,312 (3.7)	18,721 (3.5)
Arkansas	10,310 (2.3)	11,750 (2.2)
California	84,653 (19.1)	96,830 (18.1)
Florida	41,298 (9.3)	54,755 (10.3)
Georgia	31,543 (7.1)	32,481 (6.1)
Louisiana	23,031 (5.2)	25,568 (4.8)
Minnesota	11,389 (2.6)	16,844 (3.2)
Mississippi	17,284 (3.9)	18,888 (3.5)
New York	70,602 (16.0)	97,243 (18.2)
North Carolina	40,180 (9.1)	41,878 (7.8)
Pennsylvania	10,374 (2.3)	18,615 (3.5)
South Carolina	15,134 (3.4)	20,439 (3.8)
Tennessee	32,329 (7.3)	34,784 (6.5)
Texas	37,985 (8.6)	44,823 (8.4)
**Medical conditions^c^**		
Bariatric surgery	5,158 (1.0)	6,726 (1.1)
Breast cancer	11,072 (2.1)	13,016 (2.1)
Diabetes	159,042 (30.4)	190,648 (30.1)
Endometrial and ovarian cancer	2,259 (0.4)	2,557 (0.4)
Epilepsy	43,213 (8.3)	55,666 (8.8)
Malignant gestational trophoblastic disease	118 (0.0)	123 (0.0)
Human immunodeficiency virus	23,865 (4.6)	22,894 (3.6)
Hypertension	207,286 (39.7)	259,571 (40.9)
Ischemic heart disease	12,357 (2.4)	13,577 (2.1)
Liver cancer	273 (0.1)	342 (0.1)
Lupus	15,750 (3.0)	20,014 (3.2)
Schistosomiasis	120 (0.0)	—d
Solid organ transplant	588 (0.1)	578 (0.1)
Peripartum cardiomyopathy	2,817 (0.5)	3,024 (0.5)
Sickle cell disease	8,395 (1.6)	9,564 (1.5)
Severe cirrhosis	6,626 (1.3)	9,451 (1.5)
Stroke	8,090 (1.5)	9,612 (1.5)
Thrombogenic heart disease	4,944 (0.9)	5,645 (0.9)
Tuberculosis	2,938 (0.6)	2,469 (0.4)
Valvular heart disease	7,645 (1.5)	8,630 (1.4)

a Values are number (percentage) unless otherwise indicated. Percentage is the study population (women with a high-risk condition) relative to the overall population. Denominators of percentages vary because some women had more than one disorder.

b The Southeastern states were chosen as a focal point because of their similarity to each other in contraception health policy (12) and spending levels for Medicaid (13). In addition, the health rankings of these states are among the lowest in the country (14). The other 4 states chosen (California, Minnesota, New York, and Pennsylvania) were highly populous states from regions of the country that have various health policies and reimbursement levels that represent differences across the United States.

c Percentage is stratification group relative to sum of women in that strata. The sum of all categories in the stratification group may be greater than the total study population; women can belong to more than 1 category in the same stratification. Medical conditions totals used are 522,556 and 634,107, before and after MEC phases respectively. Medical conditions are the 20 disorders identified in the 2010 Centers for Disease Control and Prevention’s US Medical Eligibility Criteria for Contraceptive Use that increase risk for adverse outcomes in pregnancy (1).

d Total population was fewer than 11. The Centers for Medicare and Medicaid Services data use agreement does not allow publication of results when study population (eg, patients) is fewer than 11 participants.

### Outcome analysis

**Figure 1:**
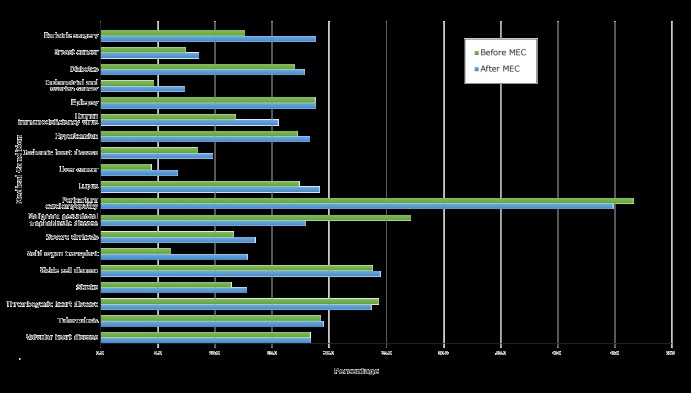
Changes in percentage of women, by medical condition, with a Medicaid claim for family planning management from the 2-year period before (2008 and 2009) to the 2-year period after (2011 and 2012) the 2010 release of the US Medical Eligibility Criteria for Contraceptive Use (MEC) by the Centers for Disease Control and Prevention (1). Percentage is number of women with each medical condition and an FPM Medicaid claim relative to the total population for that condition.

**FPM outcome: aggregate and condition-level analysis. **Provision of FPM for all reproductive-aged women in Medicaid increased from 17.9% before MEC to 18.2% after MEC. We saw a comparable increase for women in the study population, from 16.7% before MEC to 17.8% after MEC (Table 2) (estimate: 1.06; lower bound CI: 1.05). Provision of FPM varied by medical condition, ranging from 4.4% before MEC and 6.7% after MEC for those with liver cancer to 46.6% before MEC and 44.8% after MEC for those with peripartum cardiomyopathy. The conditions with the highest rates of FPM provision for both periods were peripartum cardiomyopathy, sickle cell disease, and thrombogenic heart disease (Figure 1). Gestational trophoblastic disease had the second highest provision of FPM before MEC.

**Table 2:** Provision of Family Planning Management (FPM) for Reproductive-Aged Women with Medical Conditions Enrolled in Medicaid in the 2-Year Period Before (2008 and 2009) and 2-Year Period After (2011 and 2012) the 2010 Release of the US Medical Eligibility Criteria for Contraceptive Use (MEC)

Family Planning Management^a^	Total Before MEC, 2008–2009^a^	FPM Provision Before MEC, 2008–2009^a^	Total After MEC, 2011–2012^a^	FPM Provision After MEC, 2011–2012^a^	Estimate^b^ (Lower Bound CI^c^)	P Value^d^
**Overall population**	12,422,899	2,221,325 (17.9)	13,597,612	2,477,023 (18.2)	NA	NA
**Study population**	437,018	87,115 (16.7)	527,660	112,851 (17.8)	1.06 (1.05)	<.001
**Medical conditions**						
Bariatric surgery	5,158	650 (12.6)	6,726	1,265 (18.8)	1.49 (1.42)	<.001
Breast cancer	11,072	822 (7.4)	13,016	1117 (8.6)	1.16 (1.13)	<.001
Diabetes	159,042	26,915 (16.9)	190,648	33,928 (17.8)	1.05 (1.04)	<.001
Endometrial and ovarian cancer	2,259	105 (4.6)	2,557	187 (7.3)	1.58 (1.48)	<.001
Epilepsy	43,213	8,104 (18.8)	55,666	10,469 (18.8)	1.00 (0.98)	.55
Human immunodeficiency virus	23,865	2,816 (11.8)	22,894	3,549 (15.5)	1.31 (1.28)	<.001
Hypertension	207,286	35,681 (17.2)	259,571	47,465 (18.3)	1.07 (1.06)	<.001
Ischemic heart disease	12,357	1,049 (8.5)	13,577	1,331 (9.8)	1.15 (1.12)	<.001
Liver cancer	273	12 (4.4)	342	23 (6.7)	1.52 (1.25)	<.001
Lupus	15,750	2,731 (17.3)	20,014	3,830 (19.1)	1.10 (1.07)	<.001
Malignant gestational trophoblastic disease	118	32 (27.1)	123	22 (17.9)	0.66 (0.49)	.99
Peripartum cardiomyopathy	2817	1,312 (46.6)	3.024	1,355 (44.8)	0.96 (0.90)	.95
Severe cirrhosis	6626	769 (11.6)	9,451	1,275 (13.5)	1.16 (1.12)	<.001
Sickle cell disease	8,395	1,996 (23.8)	9,564	2,337 (24.4)	1.03 (1.00)	.02
Solid organ transplant	588	36 (6.1)	9,612	74 (12.8)	2.14 (1.86)	<.001
Stroke	8,090	922 (11.4)	5,645	1,224 (12.7)	1.12 (1.08)	<.001
Thrombogenic heart disease	4,944	1,200 (24.3)	2,469	1,335 (23.6)	0.97 (0.93)	.94
Tuberculosis	2,938	564 (19.2)	8,630	481 (19.5)	1.02 (0.96)	.24
Valvular heart disease	7,645	1,399 (18.3)	9,612	1,584 (18.4)	1.00 (0.96)	.52

Abbreviation: CI, confidence interval; NA, not applicable.

a Values are number (percentage). Percentage is number of women with an FPM claim relative to women in that disease category. FPM claim includes all claims with an ICD-9 (*International Classification of Disease, Ninth Revision*)(17) code that begins with V25.

b The estimate is the ratio of the after-MEC scaled rate to the before-MEC scaled rate. A ratio greater than 1 indicates an increase in provision; a ratio of 1.1 indicates a 10% increase in the rate.

c 1-sided 99% confidence interval.

d *P *values are based on 1-sided Poisson test at a 99% confidence level. Bonferroni adjustment for *P *value threshold is .003.

**Table:** 

MEC Medical Condition	Before (2008-2009), %	After (2011-2012), %
Bariatric surgery	12.6	18.8
Breast cancer	7.4	8.6
Diabetes	16.9	17.8
Endometrial and ovarian cancer	4.7	7.3
Epilepsy	18.8	18.8
Malignant gestational trophoblastic disease	27.1	17.9
Human immunodeficiency virus	11.8	15.5
Hypertension	17.2	18.3
Ischemic heart disease	8.5	9.8
Liver cancer	4.4	6.7
Lupus	17.3	19.1
Solid organ transplant	6.1	12.8
Peripartum cardiomyopathy	46.6	44.8
Sickle cell disease	23.8	24.4
Severe cirrhosis	11.6	13.5
Stroke	11.4	12.7
Thrombogenic heart disease	24.3	23.6
Tuberculosis	19.2	19.5
Valvular heart disease	18.3	18.4

Before and after the MEC release, 12 of the 19 conditions examined showed a significant increase at the 1% significance level. After accounting for the increase at the overall population level, 5 conditions showed a greater than 30% increase in FPM: bariatric surgery, endometrial and ovarian cancer, HIV, liver cancer, and solid organ transplant (Table 2). The 7 conditions that did not show a significant increase in FPM were epilepsy, malignant gestational trophoblastic disease, peripartum cardiomyopathy, sickle cell disease, thrombogenic heart disease, tuberculosis, and valvular heart disease (Table 2).

**Figure 2:**
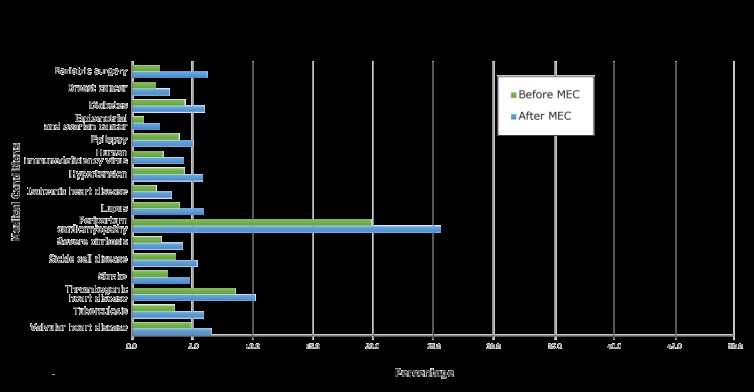
Changes in percentage of women, by medical condition, with a Medicaid claim for a highest efficacy contraception method from the 2-year period before (2008 and 2009) to the 2-year period after (2011 and 2012) the 2010 release of the US Medical Eligibility Criteria for Contraceptive Use (MEC) by the Centers for Disease Control and Prevention (1). Highest efficacy methods are contraceptive implants, intrauterine devices, and sterilization. Percentage is number of women with each medical condition and an HEM Medicaid claim relative to the total population for that condition.

**HEM outcome: aggregate and condition-level analysis.** Of the 12,422,899 reproductive-aged women insured by Medicaid, 437,036 had a HEM claim (3.5% ) before MEC’s release; 679,230 of the 13,597,612 women (5.0%) insured by Medicaid had a HEM claim after MEC (Table 3) (estimate: 1.37; lower bound CI: 1.36). We saw a comparable increase for women in the study population, from 4.1% to 5.7%. Provision of HEM varied by medical condition in both periods, ranging from 0.9% before MEC for endometrial or ovarian cancer to 25.6% after MEC for peripartum cardiomyopathy (Figure 2). After accounting for the increase at the overall population level, all 16 conditions showed a significant increase at the 1% significance level (Table 3). HEM provision more than doubled for 2 conditions: bariatric surgery and endometrial and ovarian cancer.

**Table 3:** Provision of Highest Efficacy Contraception Methods (HEM)^a^ for Reproductive-Aged Women with Medical Conditions Enrolled in Medicaid in the 2-Year Period Before (2008 and 2009) and 2-Year Period After (2011 and 2012) the 2010 Release of the US Medical Eligibility Criteria for Contraceptive Use (MEC)

Conditions Requiring Highest Efficacy Methods	Total Before MEC, 2008–2009^a^	HEM Provision Before MEC, 2008–2009^a^	Total After MEC, 2011–2012^a^	HEM Provision After MEC, 2011–2012^a^	Estimate^b^ (Lower Bound CI^c^)	*P* Value^d^
Overall population	12,422,899	437,036 (3.5)	13,597,612	679,230 (5.0)	NA	NA
Study population	437,018	21,413 (4.1)	527,660	36,176 (5.7)	1.37 (1.36)	.001
Bariatric surgery	5,158	114 (2.2)	6,726	416 (6.2)	2.8 (2.68)	.001
Breast cancer	11,072	214 (1.9)	13,016	400 (3.1)	1.59 (1.55)	.001
Diabetes	159,042	6,892 (4.3)	190,648	11,377 (6.0)	1.38 (1.37)	.001
Endometrial and ovarian cancer	2,259	21 (0.9)	2,557	58 (2.3)	2.43 (2.27)	.001
Epilepsy	43,213	1,658 (3.8)	55,666	2,813 (5.1)	1.32 (1.30)	.001
Human immunodeficiency virus	23,865	602 (2.5)	22,894	976 (4.3)	1.69 (1.65)	.001
Hypertension	207,286	8,902 (4.3)	259,571	15,072 (5.8)	1.35 (1.34)	.001
Ischemic heart disease	12,357	242 (2.0)	13,577	439 (3.2)	1.65 (1.60)	.001
Lupus	15,750	615 (3.9)	20,014	1,187 (5.9)	1.52 (1.48)	.001
Peripartum cardiomyopathy	2,817	559 (19.8)	3,024	775 (25.6)	1.29 (1.21)	.001
Severe cirrhosis	6,626	159 (2.4)	9,451	391 (4.1)	1.72 (1.66)	.001
Sickle cell disease	8,395	302 (3.6)	9,564	511 (5.3)	1.49 (1.44)	.001
Solid organ transplant	588	—e	578	24 (4.2)	NA	NA
Stroke	8,090	237 (2.9)	9,612	452 (4.7)	1.60 (1.55)	.001
Thrombogenic heart disease	4,944	423 (8.6)	5,645	576 (10.2)	1.19 (1.14)	.001
Tuberculosis	2,938	102 (3.5)	2,469	146 (5.9)	1.71 (1.61)	.001
Valvular heart disease	7,645	371 (4.9)	8,630	563 (6.5)	1.35 (1.30)	.001

Abbreviations: CI, confidence interval; NA, not applicable.

a Values are number (percentage). Percentage is number of women with an HEM Medicaid claim relative to the population in that disease category. HEM claims for contraception are for intrauterine devices, contraceptive implants, and sterilization.

b Estimated ratio of the after-MEC scaled rate to the before-MEC scaled rate. A ratio greater than 1 indicates an increase in provision; a ratio of 1.1 indicates a 10% increase in the rate.

c 1-sided 99% confidence interval.

d *P* values are based on 1-sided Poisson test at 99% confidence level. Bonferroni adjustment for *P* value threshold is *P* < .006.

e Total population was fewer than 11. The Centers for Medicare and Medicaid Services Data Use Agreement does not allow publication of results when study population (eg, patients) is fewer than 11 participants.

**Table:** 

MEC Medical Condition	Before MEC (2008 and 2009), %	After MEC (2011 and 2012), %
Bariatric surgery	2.2	6.2
Breast cancer	1.9	3.1
Diabetes	4.3	6.0
Endometrial and ovarian cancer	0.9	2.3
Epilepsy	3.8	5.1
Human immunodeficiency virus	2.5	4.2
Hypertension	4.3	5.8
Ischemic heart disease	2.0	3.2
Lupus	3.9	5.9
Peripartum cardiomyopathy	19.8	25.6
Sickle cell disease	3.6	5.3
Severe cirrhosis	2.4	4.1
Stroke	2.9	4.7
Thrombogenic heart disease	8.6	10.2
Tuberculosis	3.5	5.9
Valvular heart disease	4.9	6.5

## Discussion

Our study showed an overall increase in provision of FPM and HEM from the 2-year period before MEC’s release to the 2-year period after its release for women with 1 or more of the 20 medical conditions MEC identified as high risk for pregnant women. When all conditions were considered together, the difference was significant for both FPM and HEM. For individual conditions, significance was found for FPM for most medical conditions and for HEM for all medical conditions. The increase in HEM provision mirrors national trends. According to an analysis by the National Survey of Family Growth, the use of IUDs and contraceptive implants among reproductive-aged American women increased from 6% in 2008 to 12% in 2012 (18). Although our study accounted for the increase seen in the overall population and documented an increase across medical conditions, HEM rates for women with 1 or more of the 20 conditions were below the national average. Champaloux and colleagues had a similar finding in their review of claims of women with medical conditions from a privately insured population (19).

HEMs are particularly important for women with MEC-identified medical conditions, because pregnancy can result in severe adverse health outcomes for this population. The physiologic changes of pregnancy affect nearly every organ system in the body. For example, normal pregnancy creates a state of anemia, increased oxygen demand and cardiac output, hypercoagulability, immune compromise, and insulin resistance. These necessary changes support gestation and are generally well tolerated by healthy women. However, women with underlying medical conditions may experience amplification of their condition or predisposition to complications and illness, including death (20). The maternal death rate in the United States is the highest in the developed world (21). A recent review of maternal deaths from 9 states identified hemorrhage, cardiovascular and coronary conditions, infection, and cardiomyopathy as the most common causes (22). The review identified age-related differences underlying the cause of death and estimated that 63.2% of these deaths were preventable. One step proximal to preventing maternal death is preventing maternal illness. To prevent increased risk associated with pregnancy, a woman with a high-risk medical condition should have ready access to the most effective methods of contraception until she desires pregnancy. Then, when planning to conceive, a woman should have access to preconception care to optimize her health, manage medications, and transition her to and through pregnancy. This approach will help women with high-risk conditions to attain their reproductive goals while decreasing their health risk (23).

Our study found that provision of FPM and HEM varied by medical condition. For example, we found lower rates for FPM and HEM relative to other medical conditions among women with cancer (breast, endometrial, and ovarian cancer). One explanation is the nature of these conditions and the methods used to treat them. For example, hysterectomies or bilateral oophorectomies are common forms of treatment for endometrial and ovarian cancer, eliminating the need for contraception. Where the ability to conceive remains intact, cancers can limit women’s contraceptive options. For example, IUDs are contraindicated for women with endometrial cancer as are hormonal IUDs and implants for women with breast cancer (1). On the other hand, we found that peripartum cardiomyopathy had the highest rates of FPM and HEM. One possible reason is that this condition is associated with high rates of illness and death rates as high as 14% for a subsequent pregnancy (24). Second, by definition, peripartum cardiomyopathy is diagnosed in the last month of pregnancy or the first few months after delivery. The timing of the diagnosis may create the opportunity for a health care provider to educate a woman on the importance of contraception because of the high risk associated with a subsequent pregnancy. However, the medical conditions affecting most women fall in between these extremes. Hypertension, diabetes, epilepsy, and HIV affected more than 430,000 women in our study, and these conditions also put women at high risk for adverse health outcomes with pregnancy. Therefore, additional focus should also be placed on these conditions.

Our study had several limitations. CMS data restricted us to the clinician’s diagnosis and procedure coding during the visit. Therefore, we may not have captured data on women using contraception methods that did not require a clinician or using methods for uncoded services. For example, we may not have captured data on women with previously placed IUDs or implants if surveillance of these devices was not coded during an annual or other visit. Similarly, we were only able to reliably capture data on sterilization procedures that occurred during the years of our study. Hence, data were not captured on women who used tubal sterilization and partner vasectomy as a form of birth control. For these reasons, we believe our findings to be underestimates. Because claims data do not include sexual or relationship history, we were unable to ascertain whether a woman was at risk for pregnancy on the basis of sexual activity with a male partner, nor were we able to assess whether her medical condition precluded sexual activity or fertility. Medicaid eligibility criteria for women vary by state, and women who become pregnant may be eligible for Medicaid for a limited amount of time. For 2 common conditions, hypertension and diabetes, MEC guidelines apply to women with severe disorders; our analysis was more inclusive by showing all women with the disorders. Finally, our statistical analysis shows associations but cannot directly address causality or reasons for a change.

Overall, our study found a limited, but encouraging, change in clinical practice in the 2 years after the release of MEC guidelines. The relatively low rate of FPM and provision of HEM that we found suggests that access to highly effective contraceptives was a barrier. Access issues for contraception can arise from financial and systems issues as well as from provider bias (25). Such barriers may also present opportunities for ongoing and future steps toward full implementation of MEC guidelines.

Historically, access to contraception has been limited, especially for low-income women (26). Several efforts were made to lessen financial and system barriers to accessing contraception after the 2010 release of the MEC. After the Affordable Care Act mandate for contraceptive coverage went into effect, the percentage of women using IUDs and implants increased among sexually active women, whereas the use of oral contraception remained flat (27). The 6|18 Initiative (28) of CDC and its partners outlined 4 interventions for reducing financial and logistic barriers for public and private payers and providers. For women with no insurance coverage, family planning services can be obtained from the Federal Title X grant (29). These multilevel and collaborative approaches to reducing barriers may serve to increase the uptake of the MEC guidelines (30). In addition to these interventions, parallel programs have been working to ensure provider knowledge and application of MEC in practice. These include endorsement and implementation support of MEC by several medical associations, including the American College of Obstetricians and Gynecologists and the American Academy of Family Physicians (31,32). Focusing future efforts on specialist health care providers may help ensure that women with high-risk medical conditions receive evidence-based care and referrals to contraception counseling.

## Appendix A ICD-9^a^ Codes for 20 High-Risk Medical Conditions Identified by the US Medical Eligibility Criteria for Contraceptive Use

**Table:**

Condition	ICD-9 Code
Breast cancer	174
Diabetes	250
Endometrial and ovarian cancer	179, 182, 183
Epilepsy	345
History of bariatric surgery (past 2 years) ^a^	V45.86
Human immunodeficiency virus	042
Hypertension	401–405
Ischemic heart disease	410, 412–414
Malignant gestational trophoblastic disease	181
Malignant liver tumors and hepatocellular carcinoma of the liver	155
Peripartum cardiomyopathy	674.5
Schistosomiasis with fibrosis of the liver	120.9
Severe cirrhosis	571
Sickle cell disease	282.6
Solid organ transplant in the past 2 years^b^	V42.0, V42.1, V42.6, V42.7, V42.83, V42.9
Stroke	430–434, 436–438
Systemic lupus erythematosus	710.0
Thrombogenic mutations	286.
Tuberculosis	010–018
Valvular heart disease	424

^a^ *International Classification of Disease, Ninth Revision *(17).

^b^ Current Procedural Terminology code; used to identify surgical medical conditions (16).

## Appendix B Poisson Ratio Test Model to Determine an Increase in Family Planning Management and Provision of the Highest Efficacy Contraception Methods

Setting up the problem, we define:

*C*_i_^k^: Total number of women in overall population in time period *i* for outcome *k*

*C*_i_ = Sum of C_i_^k^ for all k = Total overall population in time period *i*

π_i_^k^ = *Rate of outcome k in time period in for overall population = C_i_^k^ over C_i_*

*M*_i_^k^ = Total number of women in study population in time period *i* for outcome *k*

*M*_i_ = Sum of *M*_i_^k^ for all *k* = Total study population in time period *i*

Given that *M_i_* is a subset of the overall population, the expected number of women in the study population with outcome *k* is

E[*M_i_^k^*] = π_i_^k^ × *M_i_^k^*

Furthermore, let

µ^k^_ij_: *Scaled proportion of the study population for time period i and medical condition j*

where:

phase *i* ∈ (0,1)

medical condition j ∈ (1,2,…20)

outcome *k* ∈ (1,2)

We determined the rate for each time-period as:

- Outcome 1: family planning management (FPM) ratio
- Counseling, insertions, and surveillance for contraceptive methods
  μ_ij_^1^ = (scaled number of women from study population with FPM claim) over (total study population) = (M_i_^1^ − E[M_i_^1^])/M_i_
- Outcome 2: highest efficacy method (HEM) ratio
- Intrauterine device (IUD): insertion and surveillance
- Implants: insertion
  μ_ij_^2^ = (scaled number of women from study population with HEM claim)/(total study population) = (M_i_^2^ - E[M_i_^2^]) / M_i_

The ratio of rates in time period 0 and time period 1 were assessed by using a 1-sided exact Poisson test.

H_0_: (μ_1j_^k^/μ_0j_^k^) = 1

H_1_: (μ_1j_^k^/μ_0j_^k^) > 1

All analysis was completed by using R version 3.4.3 (https://www.r-project.org/).
